# Tracing *Acinetobacter baumannii*’s Journey from Hospitals to Aquatic Ecosystems

**DOI:** 10.3390/microorganisms12081703

**Published:** 2024-08-18

**Authors:** Irina Gheorghe-Barbu, Rares-Ionut Dragomir, Gratiela Gradisteanu Pircalabioru, Marius Surleac, Iulia Adelina Dinu, Madalina Diana Gaboreanu, Ilda Czobor Barbu

**Affiliations:** 1 Faculty of Biology, University of Bucharest, Intr. Portocalelor No. 1–3, 060101 Bucharest, Romania; irina.gheorghe@bio.unibuc.ro (I.G.-B.); r.dragomir20@s.bio.unibuc.ro (R.-I.D.); dinu.iulia-adelina@s.bio.unibuc.ro (I.A.D.); gaboreanu.diana-madalina@s.bio.unibuc.ro (M.D.G.); ilda.barbu@bio.unibuc.ro (I.C.B.); 2 The Research Institute of the University of Bucharest (ICUB), B.P Hasdeu No. 7, 050095 Bucharest, Romania; marius.surleac@gmail.com; 3 National Institute for Infectious Diseases, “Matei Balș”, Dr. Calistrat Grozovici No. 1, 021105 Bucharest, Romania

**Keywords:** resistant *Acinetobacter baumannii* isolates, wastewater and surface water microbial load, surface water microbiome, pangenome analysis, southern Romanian cities

## Abstract

Background: This study provides a comprehensive analysis of *Acinetobacter baumannii* in aquatic environments and fish microbiota by integrating culture-dependent methods, 16S metagenomics, and antibiotic resistance profiling. Methods: A total of 83 *A. baumannii* isolates were recovered using culture-dependent methods from intra-hospital infections (IHI) and wastewater (WW) and surface water (SW) samples from two southern Romanian cities in August 2022. The antibiotic susceptibility was screened using disc diffusion, microdilution, PCR, and Whole Genome Sequencing assays. Results: The highest microbial load in the analyzed samples was found in Glina, Bucharest, for both WW and SW samples across all investigated phenotypes. For Bucharest isolates, the resistance levels corresponded to fluoroquinolones > aminoglycosides > β-lactam antibiotics. In contrast, *A. baumannii* from upstream SW samples in Târgoviște showed the highest resistance to aminoglycosides. The *bla*_OXA-23_ gene was frequently detected in IHI, WW, and SW isolates in Bucharest, but was absent in Târgoviște. Molecular phylogeny revealed the presence of ST10 in Târgoviște isolates and ST2 in Bucharest isolates, while other minor STs were not specifically correlated with a sampling point. Using 16S rRNA sequencing, significant differences in microbial populations between the two locations was identified. The low abundance of Alphaproteobacteria and Actinobacteria in both locations suggests environmental pressures or contamination events. Conclusions: These findings indicate significant fecal contamination and potential public health risks, emphasizing the need for improved water quality monitoring and management.

## 1. Introduction

Increasing concerns about antibiotic resistance (AR) have led numerous groups of researchers to inquire about the effects of antibiotic-resistant bacteria (ARB) and antibiotic resistance genes (ARGs) on human and animal health, agriculture, food production, and waste management from the perspective of the One Health concept [1]. This aims to ensure optimal health for people, animals, and the environment by addressing the spread of emerging infectious diseases at their interface. It involves understanding and managing interactions among ecological, geographic, and human activity domains [2].

Wastewater treatment plants (WWTPs) and hospital environments, due to their high microbial load, have become reservoirs for ARGs and hotspots for the dissemination of AR into the environment. Conventional mechanical and biological wastewater treatment processes cannot completely eliminate all pollutants, leading to the release of pollutants into surface water bodies along with treated wastewater. Additionally, the disposal of waste and treated water from urban areas and the use of treated wastewater for irrigation fields and discharging effluent from the WWTPs into the natural ecosystems further increase the presence of resistance genes in surface water [3,4]. During wastewater treatment, surplus sludge is produced, which highlights a high diversity of microorganisms, including pathogenic ones. 

In recent years, international authorities have made significant efforts to enhance the monitoring of ARBs and ARGs across various environments. A key strategy in these efforts involves mapping the distribution of MDR nosocomial pathogens in different clinical settings and WWTPs [5,6]. Amongst these, *A. baumannii* is a renowned opportunistic nosocomial pathogen responsible for a wide range of infections, with pneumonia and septicemia being the most common. It is included in the World Health Organization’s (WHO) priority list for research on drug-resistant bacteria and AR [7,8]. In 2022, Romania reported very high levels of resistance in *A. baumannii* to fluoroquinolones, aminoglycosides, and carbapenems (ranked fourth and third, respectively, after countries such as Croatia, Greece, Cyprus, and Italy), according to the ECDC [9]. Moreover, the presence of MDR *A. baumannii* strains in wastewaters and surface waters is well documented [5,10,11,12,13]; but, despite the ubiquitous nature of *A. baumannii*, it was not associated with fish microbiota [14,15]. 

In this context, this study aims to reveal the extent of the spread of *Acinetobacter baumannii* in aquatic environments and fish microbiota by integrating culture-dependent methods and 16S metagenomics, as well as the antibiotic resistance profile of aquatic isolates and their similarities with clinical isolates.

## 2. Materials and Methods

### 2.1. Water Sampling Campaign

Two WWTPs were selected to represent different pollution sources: urbanized city, wastewater discharges in Bucharest, capital city of Romania, and, respectively, anthropogenic activities and animal waste from a dog shelter in Târgoviște.

The Glina WWTP near Bucharest, capital city of Romania, serves about 2 million inhabitants and is the largest treatment plant in Romania and one of the largest in Europe.

By 2040, it aims to process over one million cubic meters of wastewater daily. The plant’s capabilities were significantly upgraded, including new pre-treatment and biological treatment lines with capacities of 4.5 m^3^/s and 8.9 m^3^/s, respectively [16,17]. Additionally, secondary clarifiers were added, and sludge dewatering facilities were expanded. A sludge incineration plant was also built to process 173 tons of dry sludge per day, utilizing energy recovery technology to improve sustainability [18]. 

The Târgoviște WWTP serves a population equivalent of 125,000 inhabitants from Târgoviște and its surrounding areas in southern Romania. The plant is capable of handling a maximum daily flow rate of 38,386 cubic meters under dry weather conditions [19,20]. The Târgoviște WWTP utilizes a comprehensive wastewater treatment process, including preliminary treatment, primary clarification, and advanced biological treatment for nitrogen and phosphorus removal. Chemical phosphorus removal is also implemented to boost efficiency. The sludge treatment process involves the gravity thickening of primary sludge, mechanical thickening of excess sludge, anaerobic digestion, biogas cogeneration, and mechanical dewatering of digested sludge, producing dewatered sludge for disposal or reuse [20].

On 1 August 2022 and, respectively, 9 August 2022, 2 liters of wastewater and surface water samples were collected from two WWTPs in southern Romania: Glina (n = 4 samples), which collects wastewater from Bucharest (the capital city with 1.72 million inhabitants), and Târgoviște (n = 4 samples, having 79,610 inhabitants). Samples were taken from the influent, IN; activated sludge, AS, from the aeration tank; effluent, EF, of both locations; and, respectively, upstream, UP (200 m), and downstream, DO (200 m), regions of the sampled WWTPs (Dâmbovița and Ialomița rivers, respectively) and transported at 4 °C to the microbiology laboratory of the Faculty of Biology, University of Bucharest, Romania.

### 2.2. Strain Isolation, Quantification, Identification, and Antimicrobial Susceptibility Profiles

The diluted samples up to a factor of 10^−5^ were processed by membrane filtration method. The filters were inoculated on chromogenic media (CHROMagar Acinetobacter, Paris, France) and on chromogenic media supplemented with carbapenem, cephalosporin, and polymyxin antibiotics (CHROMagar CARBA; CHROMagar ESBL; and CHROMagar Colistin, Paris, France). They were incubated at 37 °C for 24 h under aerobic conditions followed by determination of the colony-forming unit number (CFU/100 mL) belonging to *A. baumannii* and to the Gram-negative non-fermenting bacilli (NF-GNB), considering filters with a number of white colonies ≤ 200 per culture medium and using the following equation:

$$D=\frac{\sum N_{1}\ldots N_{4}}{\sum V_{1}\ldots V_{4}}=\frac{Ntot}{Vtot}*100$$

where D—density or microbial load; N—total number of the colonies; V—volume × dilution.

The confirmation of carbapenemase (CP) and extended-spectrum β-lactamase (ESBL)-producing isolates and, respectively, colistin-resistant ones was performed by culturing up to 6 colonies for each phenotype on the same type of culture media, followed by taxonomic identification using MALDI-TOF MS (Bruker system, Bremen, Germany). The isolates were preserved on broth media (Mueller Hinton, Liofilchem, Roseto degli Abruzz, Italy) supplemented with 20% glycerol at −80 °C.

Ten days prior to water sampling, 17 *A. baumannii* clinical isolates were collected from the main infectious diseases hospital in Bucharest, which receives patients nationwide and discharges wastewater (after chlorination treatment) into the city’s WWTPs. The clinical samples, including bronchial secretions, wound secretions, peritoneal fluids, and rectal swabs, were cultured on blood agar and Cystine Lactose Electrolyte Deficient (CLED) agar and identified using automated systems (VitekII Compact).

The selection criteria encompassed resistance to carbapenems, cephalosporins, and colistin, along with the overall population structure of *A. baumannii*, including antibiotic-sensitive strains from aquatic environments for comparative analysis. Additionally, the study focused on the most frequently diagnosed nosocomial infections (bronchial secretion, wound secretion, peritoneal fluid, and rectal swabs) within the selected medical setting.

A total number of 83 *A. baumannii* isolates recovered from aquatic and clinical samples were tested for antibiotic susceptibility using the standard disc diffusion method, following the protocols outlined in the current editions of the Clinical and Laboratory Standards Institute (CLSI) guidelines pertinent to the isolation year [21]. The antibiotic susceptibility profiles of these isolates were tested with the following antibiotics: amikacin (30 µg); ampicillin-sulbactam (20 µg); aztreonam (30 µg); cefepime (30 µg); ceftazidime (30 µg); ciprofloxacin (5 µg); doripenem (10 µg); imipenem (10 µg); meropenem (10 µg); gentamicin (10 µg); and minocycline (30 µg). The antibiotic susceptibility results were interpreted according to the antibiotic classes, respectively, for β-lactams, fluoroquinolones, aminoglycosides, and tetracyclines.

Colistin susceptibility was determined according to the CLSI (2022) using the microdilution method in Cation-Adjusted Mueller–Hinton Broth medium (CAMHB, OXOID, Basingstoke, UK), where colistin sulfate (19.000 IU/mg, Sigma-Aldrich, Merck, Darmstadt, Germany) concentration ranged between 128 and 0.25 μg/mL. The validity of the testing was confirmed using *P. aeruginosa* ATCC 27853 and *E. coli* ATCC 13846 strains. The positive (untreated cultures) and negative controls (sterility control) were included and the minimum inhibitory concentration (MIC) values were determined after incubating for 24 h at 37 °C as being the last concentration for which no growth was recorded.

### 2.3. Characterization of Genotypic Resistance Profiles

The presence of carbapenem- and cephalosporin-encoding genes (*bla*_VIM_, *bla*_IMP_, *bla*_NDM_, *bla*_OXA-23_, *bla*_OXA-24_, *bla*_OXA-58_, *bla*_OXA-235_, *bla*_OXA-51_, *bla*_KPC_, *bla*_GES_, *bla*_SHV_, *bla*_TEM_, *bla*_CTX-M_, *bla*_PER_, and *bla*_VEB_) was investigated by simplex and multiplex PCR using a DNA template obtained using a modified alkaline extraction method, specific primers, and amplification programs and checked by gel electrophoresis [11,12].

### 2.4. Whole Genome Sequencing (WGS) and Bioinformatic Analyses of Clinical and Aquatic A. baumannii Isolates

Out of 83 *A. baumannii* isolates from two WWTPs and Fundeni Bucharest Hospital in Romania, we performed WGS on 20 isolates. The selection was based on AR profiles (covering all identified phenotypes) and isolation sources (IN, EF, AS, UP, and DO samples). Total DNA was extracted using DNeasy UltraClean Microbial Kit (Qiagen, Hilden, Germany), followed by library preparations with Nextera DNA Flex Library Prep Kit (Illumina). The sequencing was performed on Illumina MiSeq and NextSeq platforms (V3, 600 cycles).

Raw reads were assembled de novo using Shovill v1.1.0 pipeline [22], followed by annotation using Prokka v1.14.6 [23], as well as ABRicate v1.0.0 [24] tool and the NCBI and VFDB [25] databases to determinate profiles of ARGs and virulence factors (VFs). The Multilocus Sequence Type (MLST) [26] tool was used to determinate the sequence type (ST) of the isolates, in conformity with the Pasteur scheme. The output generated by Prokka served as input for Roary v3.13.0 [27]. Newick tree, generated from Roary along with core and accessory genes, was visualized using Phandango [28] online tool. Roary output was converted using a script [29] for multidimensional scaling (MDS) and pangenome tree representation by FriPan [30]. Subsequently, the Heaps’ law was determined for the data set using Seth Commichaux’s Python script [31].

### 2.5. Collection of Fish Samples

During a summer sampling campaign in August, six *Carassius gibelio* individuals (both males and females) were collected from each sampling site. This period is ideal for sampling because it coincides with the species’ spawning season and peak activity levels, and the warm, stable weather facilitates safer fieldwork. The water’s physicochemical characteristics across the lakes showed minimal variation, with temperature averaging 24 ± 1.5 °C, pH at 7.65 ± 0.5, dissolved oxygen at 3.23 ± 1.43 mg O_2_/L, chemical oxygen demand (COD) at 10 ± 1.5 mg O_2_/L, and suspended solids at 255 ± 45 mg/L. Samples were collected in sterile glass vials and stored at 4 °C in the dark. Each fish was weighed and measured before being sacrificed to collect their intestines, which were then stored in a freezer at −80 °C for future analysis. 

### 2.6. Metagenomic Analysis of Surface Water Samples and Fish Microbiota to Highlight the Connection between the Environment and Fish Microbiota

To examine the microbiome from fish gut samples, DNA extraction was performed using the Pure Link Microbiome DNA Purification Kit (Invitrogen, Thermo Scientific, Waltham, MA, USA), following the manufacturer’s instructions. DNA extraction from water samples was carried out using the DNA Power Water Kit (Qiagen, Hilden, Germany), according to the manufacturer’s instructions. The 16S rRNA sequences were then amplified using specific primer pairs for the V3–V4 hypervariable region of the 16S rRNA gene. The PCR products resulting from the amplification of the hypervariable regions of the 16S rRNA gene were purified using AmPure XP magnetic beads (Beckman Coulter, Inc., Brea, CA, USA). Library preparation was conducted using the Ion Plus Fragment Library Kit (Life Technologies, Carlsbad, CA, USA), following the manufacturer’s instructions. The obtained amplicon libraries were sequenced on an Ion Torrent 316 chip using the Ion Torrent PGM system and the Ion Sequencing 400 Kit (Life Technologies, USA), adhering to the manufacturer’s instructions. The sequencing data obtained were processed using the Quantitative Insights Into Microbial Ecology (QIIME) pipeline, a tool used for microbiome sequencing data analysis, allowing for the determination of microbiota composition and diversity. For calculating diversity measures, operational taxonomic units (OTUs) of the 16S rRNA gene were defined at a sequence similarity of at least 97%. The final analysis of the obtained sequences was performed using Ion Reporter softwar (Version 5.20.2.0).

## 3. Results

### 3.1. Phenotypic and Genotypic AR Profiles of A. baumannii Isolates

This paper analyzes *A. baumannii* in aquatic environments and fish microbiota using culture-dependent methods, 16S metagenomics, and the antibiotic resistance profiling of recent isolates from Glina, Bucharest, Târgoviște WWTP, and the receiving rivers (Dambovita and Ialomița) in southern Romania.

#### 3.1.1. Isolation and Quantification of *A. baumannii* from Romanian Wastewater and Surface Water Samples

**Figure 1:**
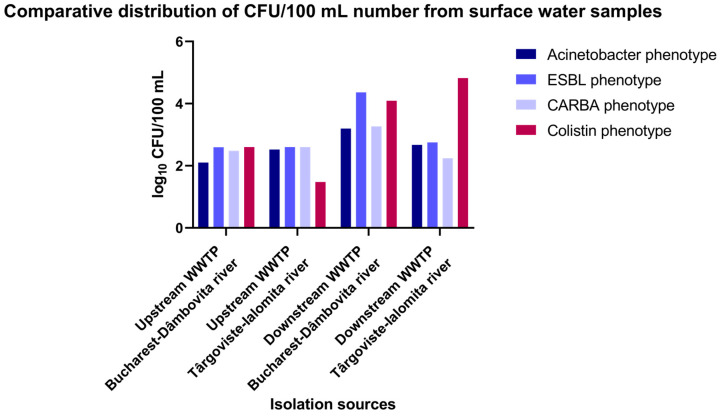
The microbial load of *Acinetobacter* for the upstream and downstream sampling points of the investigated WWTPs in the two locations in southern Romania.

**Figure 2:**
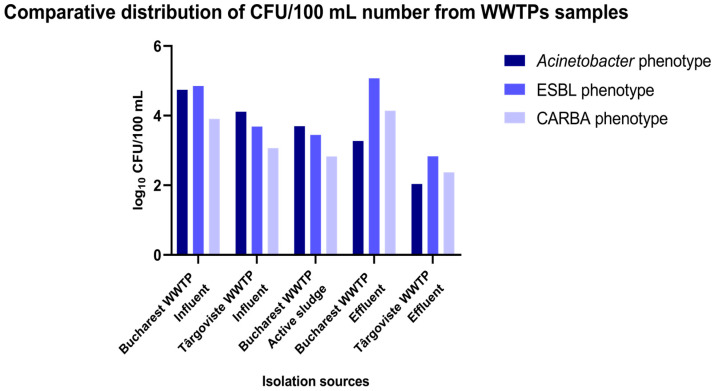
The microbial load with *Acinetobacter* for the wastewater sample collection inside the investigated WWTPs in the two locations in southern Romania.

The inoculation of diluted wastewater and surface water samples collected between 1 and 9 August 2022 from different sampling points of the investigated WWTPs (influent, active sludge, and effluent) and surface water samples collected from the upstream and downstream regions of the sampled WWTPs on chromogenic culture media (CHROMagar Acinetobacter for determining the total microbial load corresponding to *A. baumannii*) and chromogenic media supplemented with antibiotics (CHROMagar CARBA; CHROMagar ESBL; and CHROMagar Colistin) allowed for the determination of microbial load in the collected samples (Figure 1 and Figure 2; Supplementary Tables S3 and S4).

The analysis of the comparative levels of the microbial load in the analyzed samples from the receiving river are revealed in decreasing order by phenotype:

*CARBA phenotype*: DO Glina, Bucharest > UP Târgoviște > UP Glina, Bucharest > DO Târgoviște.

*Colistin phenotype*: DO Targoviste > DO Glina, Bucharest > UP Glina, Bucharest > UP Târgoviște.

*ESBL* and *total Acinetobacter phenotype*: DO Glina, Bucharest > DO Târgoviște > UP Târgoviște > UP Glina, Bucharest (Figure 1 and Supplementary Table S3).

The analysis of the comparative levels of the microbial load in the analyzed samples from the WWTPs are revealed in decreasing order by phenotype:

- *ESBL phenotype*: EF Glina, Bucharest > IN Glina, Bucharest > IN Târgoviște >AS Glina, Bucharest > EF Târgoviște.
- *CARBA phenotype:* EF Glina, Bucharest > IN Glina, Bucharest > IN Târgoviște >AS Glina, Bucharest > EF Târgoviște.
- *total Acinetobacter phenotype*: IN Glina, Bucharest > IN Târgoviște > AS Glina, Bucharest > EF Glina, Bucharest > EF Târgoviște (Figure 2 and Supplementary Table S4).

#### 3.1.2. Antimicrobial Susceptibilities Profiles of *A. baumannii* Isolates from Different Isolation Sources and by Geographical Location

**Figure 3:**
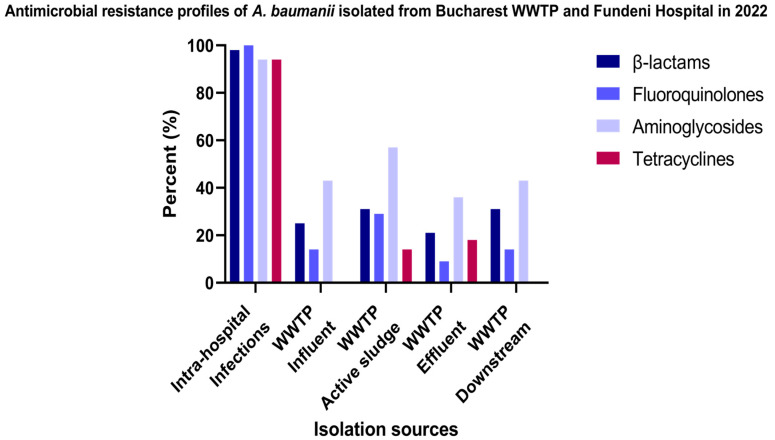
Percentage of *A. baumannii* isolates isolated from IHIs, WWTP, and surface water samples in Bucharest, categorized according to their resistance profiles to different antibiotic classes.

In decreasing order, the resistance level of *A. baumannii* isolates recovered from intra-hospital infections (n = 17) correspond to fluoroquinolones (100%) and β-lactam (98% of the isolates), followed by aminoglycosides and tetracycline antibiotics (94% of the isolates). The comparative study of the AR profiles according to the isolation sources and location demonstrated the following: for a total of 33 *A. baumannii* isolates recovered from Glina, Bucharest’s WWTP, and the receiving river, the highest resistance level corresponds to aminoglycosides in the case of the AS samples (57%), followed by the isolates isolated from EF (36%), and, respectively, the IN and DO regions (43%). For β-lactam antibiotics, the resistance levels were in decreasing order as follows: AS, DO (31% of the isolates) > IN (25%) > EF (21%). In the case of fluoroquinolone antibiotics, the resistance levels were attributed to the isolates in decreasing order as follows: AS samples (29%) > IN, DO samples (14%) > EF (9% of the isolates). The most susceptible *A. baumannii* correspond to tetracycline antibiotics (Figure 3 and Supplementary Table S5).

**Figure 4:**
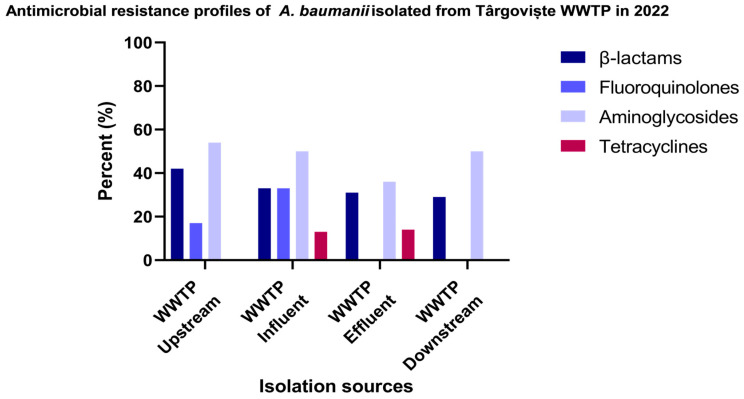
Percentage of *A. baumannii* isolates recovered from WWTP and surface water samples in Targoviste, categorized according to their resistance profiles to different antibiotic classes.

The resistance levels in the case of *A. baumannii* isolated from Târgoviște show that the aminoglycoside resistance was recorded at the top of the resistance level, with 54% of the isolates recovered from surface water sample from the UP region, followed by *A. baumannii* isolated from IN, DO (50%), and isolates isolated from the EF sample (36%). For β-lactam antibiotics, the resistance level was observed to be decreasing in the following order: surface water sample from UP region (42% of the isolates) > IN (33%) > EF (31%) and DO (29% of the isolates). The resistance levels identified for fluoroquinolone were as follows: IN samples (33%) > UP samples (17% of the isolates). The most susceptible *A. baumannii* isolates were identified for tetracycline antibiotics, as illustrated in Figure 4 and Supplementary Table S6.

Using the microdilution method in CAMHB, the MIC values were determined for colistin susceptibility as follows: in the case of one intra-hospital infection isolate (encoded 24 IHI Buc) within the international clone (IC) 2 (ST2) isolated from Fundeni hospital, it was intermediate to colistin with an MIC value of 1 μg/mL. In this study, mutations were identified in *lpxC* (N286D), *lpxD* (E117K), and *pmrB* (A138T); the genes were compared to the *A. baumannii* ATCC 1906 genome (GenBank CP045110) [32]. The rest of the clinical and wastewater isolates belonging to epidemic ICs or non-ICs were susceptible to colistin (MIC < 0.25 μg/mL) (see Table 1).

#### 3.1.3. Genotypic Characterization of β-Lactam Resistance in Clinical, Wastewater, and Surface Water *A. baumannii* Isolates

**Figure 5:**
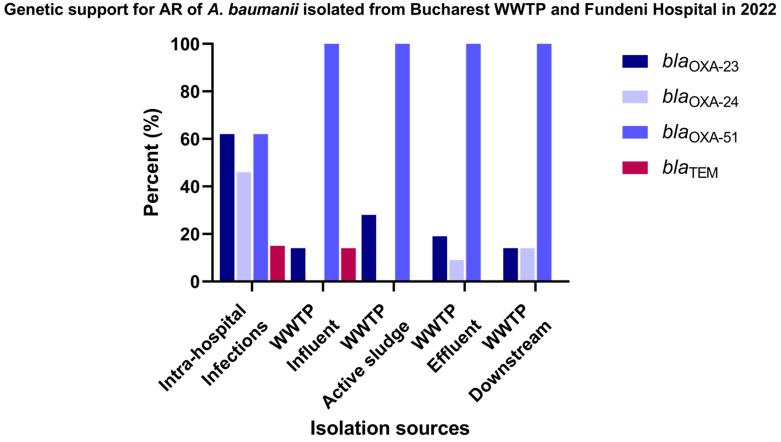
β-lactamase-producing *A. baumannii* isolates from Bucharest, Romania, in 2022.

The comparative molecular study for a total of 33 *A. baumannii* stains recovered from Glina, Bucharest, wastewater and surface water samples according to the isolation sources (Figure 5 and Supplementary Table S7) revealed that the most frequently encountered gene in investigated sources was *bla*_OXA-23_: AS (28% of the isolates) > EF (19%) and IN, DO (14% of the isolates). As anticipated, the *bla*_OXA-51_ gene was identified in all isolates isolated from all isolation sources. Nevertheless, isolates recovered from intra-hospital infections were positives for the *bla*_OXA-23_ gene (62%) and *bla*_OXA-24_ gene (46% of the isolates). Additionally, the presence of the *bla*_TEM_ gene was confirmed, although at a relatively lower prevalence (ranging from 15 to 14% in IHI and WWTP IF).

**Figure 6:**
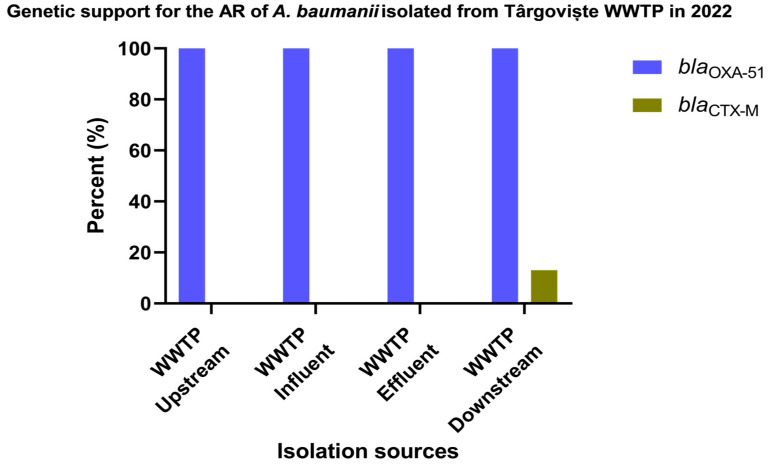
β-lactamase-producing *A. baumannii* isolates isolated from Târgoviște, Romania, in 2022.

The genotypic characterization of β-lactam resistance performed in 33 isolates of *A. baumannii* isolated from Târgoviște wastewater and surface water samples (Figure 6 and Supplementary Table S8) revealed that the *bla*_CTX-M_ gene was identified in 13% of the isolates obtained from surface water samples collected from the DO region of the sampled WWTP. As expected, the *bla*_OXA-51_ gene was identified in 100% of the isolates from all sources.

#### 3.1.4. WGS Analysis in Clinical, Wastewater, and Surface Water *A. baumannii* Isolates

The WGS analysis of intra-hospital infections and wastewater and surface water *A. baumannii* isolates revealed the presence of the following ARGs: *ant(3″)-IIa*, *aph(3″)-Ib*, *aph(3′)-VIa*, *aph(6)-Id*, and *armA* for aminoglycosides resistance. Macrolide and tetracycline resistance was confirmed by the presence of *mph(E)*, *msr(E)*, and, respectively, *tet(B)* genes. In addition, *sul1* and *sul2* genes, which encode resistance to sulfonamides, were also identified. Moreover, the genes encoding for chloramphenicol resistance, *catA1* and *cmlB1* genes, were identified in isolates from all sampled sources. Notably, *bla*_OXA-23_ *and bla*_OXA-72_ genes encoding for carbapenem resistance were present in six of the tested isolates, including isolates from intra-hospital infections and wastewater samples from Bucharest. Furthermore, in one isolate belonging to IC2, isolated from Bucharest WWTP effluent (encoded 22014-CA2), both CP-encoding genes mentioned above were identified. Nevertheless, the most prevalent genes belonged to the *bla*_ADC_ and *bla*_OXA-51_ families encoding for classes C and D β-lactamases. Additionally, the *bla*_TEM_ gene, which encodes for class A broad-spectrum β-lactamase, has also been identified in a wastewater *A. baumannii* isolate recovered from Bucharest WWTP effluent (Supplementary Table S9).

**Figure 7:**
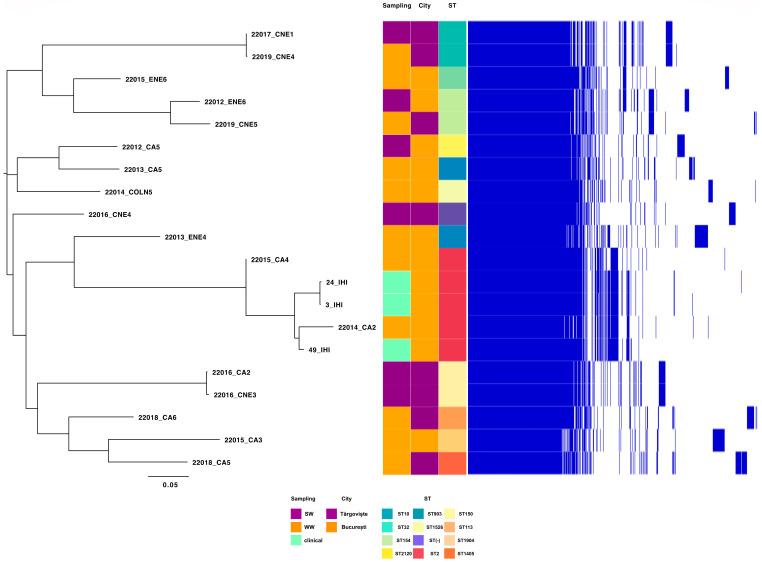
Pangenome analysis of *A. baumannii* isolates from WW, SW, and IHI samples in southern Romania based on accessory genes.

MLST analyses indicate that the most prevalent clones within the tested isolates from all isolation sources were IC2 (ST2, 25% of the sequenced isolates) followed by the IC8 (ST10, identified in the case of one isolate from wastewater and another one from a surface water sample from Targoviste) and IC7 (ST113 in the case of one wastewater isolate from Targoviste), according to Shelenkov et al.’s classification [33]. Moreover, several other non-IC clones were identified, i.e., ST154 (surface water sample from Glina, Bucharest, and wastewater isolate from Târgoviște), ST150 (surface water sample from Târgoviște), and ST32 (wastewater isolate from Glina, Bucharest) (Figure 7).

**Figure 8:**
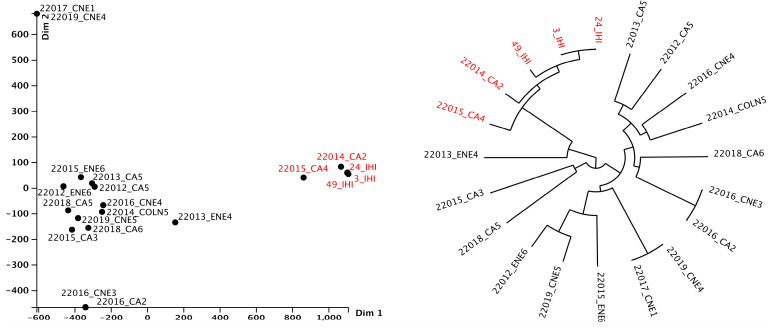
*A. baumannii* isolates’ pangenome-FriPan MDS and pangenome tree representation based on accessory genes.

Pangenome analysis was undertaken on 20 genomes of *A. baumannii* isolates from WW and SW in two cities in Romania, as well as from IHI. Notably, one cluster stands out from all 20 genomes (red color in Figure 8); it belongs to ST2 and was isolated from WW (n = 2) and IHI (n = 3) in Bucharest. The other isolates belong to various STs, such as ST150, ST10, and ST154, and correlations between isolation sources could not be established (Figure 7). Moreover, *A. baumannii* pangenome analysis identified a gene pool consisting of 7644 genes in the sequences of all 20 isolates. Most of these genes (~60%) are classified as hypothetical proteins. The 5-isolate cluster has 224 unique genes compared to the other 15 isolates in this study. Of these 224 genes, most of them are classified as hypothetical proteins (88%), but there are other genes that could be linked to antibiotic resistance (e.g., *tetA*, *tetR* (associated with Tn*10*), and other MGEs (Tn*3* family transposase Tn*2*, Tn*3* family transposase TnAs*3*, IS*66* family transposase IS*Aba17*). Following Heaps’ law, the pangenome of *A. baumannii* remains open, characterized by a γ = 0.26.

Using the VFDB database, it was shown that circulating *A. baumannii* clones in Romanian intra-hospital infections from Bucharest and wastewater from two cities in southern Romania were associated with virulence factors encoding for adherence (*ata, tufA*), biofilm formation (*algW*, *bap*, *csuA*, *csuB*, *csuC*, *csuD*, *csuE*), effector delivery systems (*tssA*, *B*, *C*, *D*, *E*, *F*, *G*, *K*, *L*, *M*, *tagX*), exoenzymes (*cpaA*), immune modulation (*cps4l*, *galE*, *galU*, *pseB*, *pseC*, *pseF*, *pseG*, *pseH*, *pseI*, *tviB*, *wbpD*), and nutrition/metabolic factors (*bauA*, *hemO*) [34]. The highest number of virulence factors were associated with clinical and wastewater isolates positives for OXA-23 and OXA23+OXA-72 CPs in the case of wastewater isolates isolated from Bucharest WWTP effluent and belonging to IC2: (40 and, respectively, 35 encoding genes). An interesting situation was observed in the case of the *cpaA* gene, identified in *A. baumannii* isolates recovered from wastewater and surface water samples from both investigated locations, and associated with ST154, ST903, ST32, and ST10 clones (Supplementary Table S10).

### 3.2. Metagenomic Analysis of Surface Water Samples and Fish Microbiota to Highlight the Connection between the Environment and Fish Microbiota

**Figure 9:**
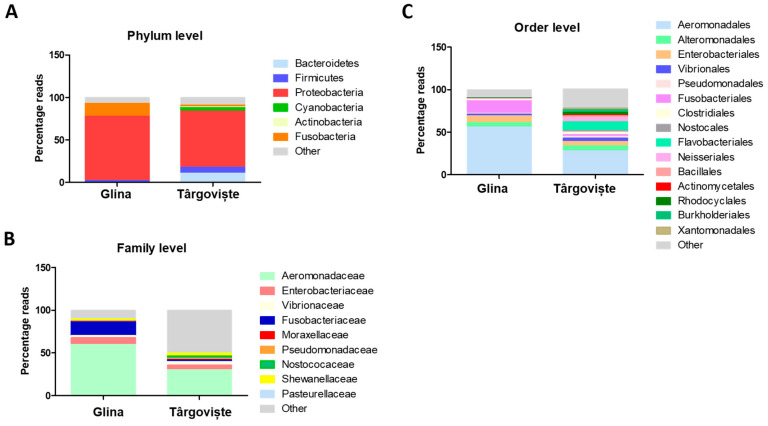
Bar graphs depicting the composition of microbial communities within fish intestines from two Glina and Târgoviște samples. The graphs show the relative abundance of various taxa at different taxonomic levels: Phylum (**A**), Order (**B**), and Family (**C**).

Figure 9 depicts the composition of microbial communities within fish intestines from two Glina and Târgoviște samples at different taxonomic levels. Proteobacteria (red) was the dominant phylum in both locations, whereas Firmicutes (blue) and Bacteroidota (light blue) are also present but in smaller proportions (Figure 9A). Other phyla, including Cyanobacteria, Actinobacteria, and Fusobacteria, are present in minor amounts. Both locations show similar phylum distributions, with a noticeable prevalence of Proteobacteria. Fish samples from Târgoviște showed a slightly higher proportion of Firmicutes compared to fish samples harvested from Glina. 

Aeromonadales (light blue) was the most dominant bacterial order in samples collected from both locations. Pseudomonadales (light pink), Vibrionales (orange), and Alteromonadales (light green) are also significant. Other orders are present in smaller proportions and show diversity across both locations. Samples from Glina showed a higher dominance of Aeromonadales. Conversely, fish samples from Târgoviște exhibited a more even distribution across different orders, indicating greater microbial diversity (Figure 9B).

The fish samples collected from Târgoviște exhibited a microbiota predominantly composed of Aeromonadales species (28%), Flavobacteriaceae (10%), Enterobacteriaceae (5%), and Vibrionaceae (4%) (Figure S1 and Figure 9C). The Alpha Proteobacteria was poorly represented, with an abundance of only 2%. The order Pseudomonadales was present in the gut samples with an abundance of 2% of the total number of sequences obtained. The microbiome at this site was predominantly composed of Aeromonadaceae species (56%) and a very high abundance of Fusobacteriaceae (15%). The percentage of Enterobacteriaceae (8%) was higher compared to that obtained for the samples collected from Târgoviște, indicating a degree of fecal contamination, as this family of microorganisms is associated with the human microbiome (Figure S1 and Figure 9).

**Figure 10:**
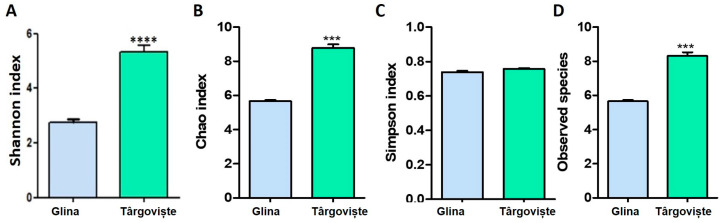
Microbiome diversity of microbial communities in fish intestine samples from Glina and Targoviste measured by Shannon index (**A**), Chao index (**B**), Simpson index (**C**), and number of observed species (**D**); *** *p* < 0.001; **** *p* < 0.0005 by Student *t*-test.

Fish samples from the Glina, Bucharest, site exhibited lower microbial diversity compared to those from Târgoviște, as measured by the Shannon, Simpson, and Chao indices, and the number of observed species (Figure 10A–D). The Shannon index, which reflects both species richness and evenness, was significantly higher in the intestines of fish from Târgoviște (Figure 10A).

The Shannon index, which accounts for both richness (the number of species) and evenness (how evenly the individuals are distributed among the species) was significantly higher in fish intestines from Târgoviște compared to Glina (Figure 10A).

The Chao index estimates the total number of species in a community, including rare species that might not be well sampled. The Chao index was also significantly higher in Târgoviște compared to Glina, suggesting that fish intestines from this location hosted a greater number of different species, including rare or less abundant species, than those in Glina (Figure 10B). 

The Simpson index measures the probability that two individuals randomly selected from a sample will belong to the same species and a lower value indicates higher diversity. In our analysis, both locations had a Simpson index close to 1, indicating a high diversity with no significant difference between the two locations. While the Shannon and Chao indices showed differences, the Simpson index suggests that the dominance structure of species (how much one species outnumbers others) might be similar in both locations (Figure 10C). 

By analyzing the number of different species observed in the sample, we observed a significantly higher number of species in fish samples from Târgoviște compared to Glina. Hence, we found a greater species richness in the microbial communities of fish intestines from Târgoviște (Figure 10D). All metrics except the Simpson index showed significantly higher diversity in the fish intestines from Târgoviște compared to Glina. 

**Figure 11:**
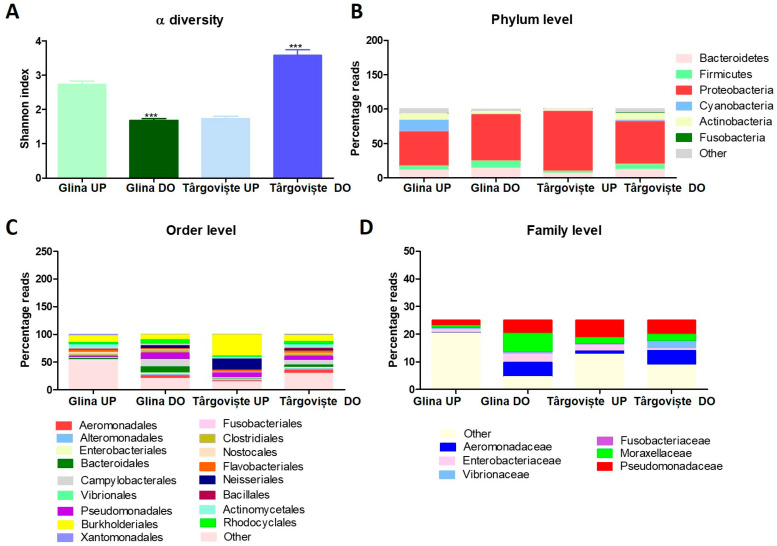
A detailed analysis of the microbiome composition in upstream (“UP”) and downstream (“DO”) water samples from Glina and Târgoviște: microbiome α-diversity (**A**), bacterial phylum level (**B**), order level (**C**), and family level (**D**).

Figure 9 and Figure S2 illustrate the composition of the microbial communities identified in water samples collected from Glina and Târgoviște. The Shannon index measures the diversity within a sample, considering both species richness and evenness. While the UP Glina samples showed moderate diversity with a Shannon index close to 3, the downstream samples displayed a significant decrease in diversity (Shannon index around 1.5), indicating a reduction in microbial diversity (Figure 11A). The UP Targoviste samples showed a similar diversity to Glina UP samples with a Shannon index around 2.5. Conversely, the Târgoviște DO samples harbored a significant increase in diversity compared to the UP sample with the highest Shannon index. 

In terms of water microbiome composition, the phylum Proteobacteria (red) was found to be dominant across all samples, whereas other phyla such as Bacteroidetes (pink), Firmicutes (light green), and Cyanobacteria (blue) were also present, but in much smaller proportions. The overall phylum distribution was relatively consistent across all samples, with Proteobacteria being the most prevalent, and we observed no dramatic shifts in phylum-level composition between the UP and DO samples in both locations (Figure 11B).

The microbial community structure at the phylum level remained stable between UP and DO in both locations, indicating that changes in diversity might be due to shifts at lower taxonomic levels. Aeromonadales (red), Pseudomonadales (purple), Vibrionales (yellow), and Burkholderiales (dark green) were among the most dominant bacterial orders identified for all tested samples (Figure 11C).

At the family level, Aeromonadaceae (blue) and Pseudomonadaceae (red) were consistently present across all samples (Figure 11D). We identified a shift in the dominance of certain families downstream, with an increase in Pseudomonadaceae, aligning with the observed decrease in diversity. In the case of samples from Târgoviște, we observed a noticeable increase in family-level diversity DO, with more balanced representation across multiple families, correlating with the increased Shannon index. The shifts in family-level composition are more pronounced than those at the phylum or order levels. The surface water samples from the UP region of Târgoviște WWTP were characterized by a very high abundance of Burkholderiales (38%), Neisseriales (19%), and Pseudomonadales (9%). Additionally, Enterobacteriales, Aeromonadales, Alteromonadales, and Rhodobacterales were identified, albeit at lower abundances. The DO samples from Targoviste harbored Actinomycetales (6%), Aeromonadaceae (5%), and Flavobacteriaceae (4%), along with a high presence of Campylobacteraceae (6%), a family of microorganisms pathogenic to humans but commensal in some animals (e.g., chickens) (Figure S2A).

In the UP water samples collected from Glina, Bucharest WWTP, 16S rRNA sequencing revealed high levels of Burkholderiales (11%), Oscillatoriales (6%), Flavobacteriales (4%), Actinomycetales (8%), and Cytophagales (3%). The DO samples were characterized by the presence of pathogenic microorganisms (Campylobacteraceae–11%) and microorganisms typical of the human microbiota such as Enterobacteriaceae, Bacteroidaceae, Prevotellaceae, and Lactobacillales. Additionally, species of the families Pseudomonadaceae (4%) and Moraxellaceae (6%) were identified (Figure S2B).

Furthermore, the analysis of the 16S rRNA metagenomic data from the surface water samples from the DO regions of Glina, Bucharest, and Târgoviște WWTPs reveals significant insights into the microbial communities present in these environments. The focus on the Moraxellaceae family, particularly the genus *Acinetobacter*, provides a detailed understanding of bacterial diversity and prevalence.

**Figure 12:**
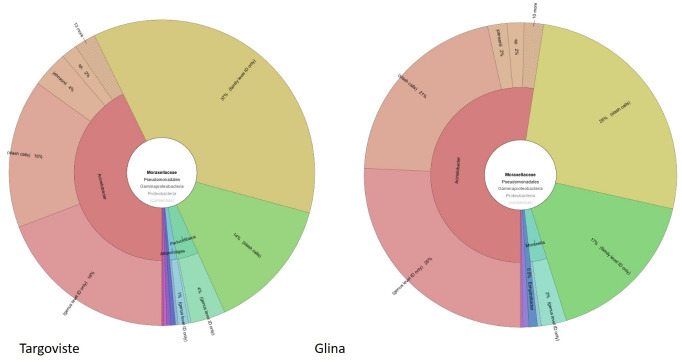
Taxonomic composition of Moraxellaceae in surface water samples from DO regions of Glina, Bucharest (**right**), and Târgoviște (**left**) WWTPs based on 16S rRNA metagenomic analysis. The inner circle represents the taxonomic classification at the family level, while the outer circle provides a more detailed view at the genus level. Glina, Bucharest: *Acinetobacter* (43%), unidentified genera within Moraxellaceae (28%), *Moraxella* (7%), *Psychrobacter* (3%), and *Enhydrobacter* (0.9%). Targoviste: *Acinetobacter* (52%), unidentified genera within Moraxellaceae (37%), *Moraxella* (7%), and *Enhydrobacter* (2%).

In the water samples collected from the DO region of Glina, Bucharest, WWTPs *Acinetobacter* was identified as a significant component of the microbial community within the Moraxellaceae family. Specifically, *Acinetobacter* constituted 43% of the Moraxellaceae reads. This substantial presence highlights the potential environmental impact and resilience of *Acinetobacter* species in this downstream water ecosystem (Figure 12).

Similarly, the water samples from Târgoviște revealed an even higher prevalence of *Acinetobacter* within the Moraxellaceae family. In this location, *Acinetobacter* accounted for 52% of the Moraxellaceae reads. This higher percentage indicates a robust population of *Acinetobacter* species in the Târgoviște DO waters (Figure 12).

## 4. Discussion

Monitoring studies of the quality parameters for five sections of the Dâmboviţa river, both upstream and downstream of Bucharest, showed that the river’s overall ecological state falls into quality classes III–V (poor to bad quality). The worst conditions corresponded to the DO region of Bucharest, which received partially treated wastewater from the Bucharest WWTP [35]. The Ialomita River’s water quality, monitored along its length, ranged from very good to very poor (classes I to V). After 2010, the water quality improved, with only the DO region showing a moderate status [36]. Accordingly, using culture-dependent assays, we revealed that the highest microbial load in the analyzed samples (wastewater from the IN, AS, and EF sources, and surface water from the UP and DO regions of the Dambovita and Ialomita rivers) was found in Bucharest for both wastewater (WWTP EF and WWTP IN) and surface water samples (DO region of the WWTP Bucharest) across all investigated phenotypes (CARBA, ESBL, colistin, and total *Acinetobacter* population). 

In our pursuit to determine the dissemination of *A. baumannii* from clinics to aquatic ecosystems, we compared the antibiotic resistance profiles of our samples. In both WWTPs, the highest resistance levels were determined for aminoglycoside antibiotics, followed by β-lactams and fluoroquinolones, with resistance levels varying by location and isolation source. Regarding IHI isolates, the resistance levels, in decreasing order, corresponded to fluoroquinolones > β-lactam > aminoglycosides and tetracycline antibiotics. The investigation of colistin resistance revealed that only one IHI isolate, belonging to ST2 and obtained from a large hospital in Bucharest that admits patients nationwide, showed intermediate resistance, while all other isolates were sensitive to colistin. Resistance to colistin may be caused by mutations in genes encoding lipopolysaccharides (LPS), such as *lpxA*, *lpxC*, and *lpxD*, as well as genes encoding for phosphoethanolamine transferase (PEtN), such as operon *pmrCAB*, as previously demonstrated [37]. Previous data have reported that ST2 is the most prevalent ST associated with colistin resistance in *A. baumannii* across Europe, Asia, Africa, and North and South America [33,38,39,40,41,42,43,44,45,46,47,48,49]. These differences in antibiotic resistance profiles between WWTPs and IHI isolates likely result from distinct environmental pressures, antibiotic usage patterns, bacterial population dynamics, horizontal gene transfer rates, and efflux pump activity.

In Romania, the most frequently detected bacterial isolates with clinical relevance include *Klebsiella* spp., *A. baumannii*, *Escherichia coli*, *Staphylococcus aureus*, and *Pseudomonas aeruginosa*, all showing the MDR phenotype. *A. baumannii*, associated with nosocomial infections such as pneumonia, meningitis, and urinary tract infections, was a focus of a 2018 study aimed at identifying microorganisms responsible for pneumonia in patients at an emergency hospital in Bucharest. Antimicrobial susceptibility testing for *A. baumannii* revealed high resistance rates: 88% to fluoroquinolones (including ciprofloxacin), 86% to β-lactam antibiotics (meropenem), and 86% to aminoglycosides (including amikacin) [50]. 

The transmission of ARGs among human, animal, and environmental reservoirs is a significant concern, with WWTPs being critical reservoirs for the spread of these genes. For *A. baumannii* isolates isolated from Romanian WWTPs, the highest resistance rates were recorded for fluoroquinolones (87.5% to ciprofloxacin), followed by aminoglycosides (86% to gentamicin and amikacin), and β-lactam antibiotics (84% to aztreonam and meropenem) [6]. Viable MDR- and carbapenem-resistant *A. baumannii* were detected in urban wastewater, which included hospital wastewater, both before and after secondary wastewater treatment [51]. Other studies have highlighted the presence of putative carbapenem-resistant *Acinetobacter* isolates detected in all WWTP samples, except the primary sludge. Also, studies have revealed that *A. baumannii* isolates were resistant to fluoroquinolones, aminoglycosides, β-lactams, and polymyxins in different sampling points of urban WWTPs [52].

The WGS analysis of *A. baumannii* isolates from investigated locations revealed both shared and unique characteristics, i.e., the *ant(3″)-IIa* gene in all isolation sources from both locations opposite to several CPs: OXA-23 (Bucharest IHI and wastewater); OXA-72 (Bucharest wastewater); OXA-23+OXA-72 (Bucharest wastewater); TEM-1 (Bucharest wastewater); OXA-121 (Târgoviște surface water); and OXA-120 (Târgoviște wastewater). In a study carried out in Croatia on *A. baumannii* isolates recovered from wastewater samples, the carbapenem-resistant isolates were positive for the *bla*_OXA-23_ gene and belonged similarly to our obtained results for IC2 and the susceptible ones for IC5. Furthermore, these isolates revealed resistance genes encoding for chloramphenicol, aminoglycosides, and tetracycline antibiotics [53]. In another study in eastern Poland, using conventional methods and metagenomic assays demonstrated the presence of *Acinetobacter* spp. and *A. baumannii* isolates carrying MBL (VIM2, NDM, and IMP-1)) and class D β-lactamases (OXA-23, OXA-24, OXA-51, OXA-58) in wastewater and river water samples collected in June and September in 2019. The high frequency of isolation of *A. baumannii* in IHI, positive for OXA-23 CP and belonging to ST2, was also described in two Bulgarian hospitals, Romania’s neighboring country. The CHLDs linked to IC2 were also reported in clinical *A. baumannii* in other neighboring countries of Romania: OXA-23 and OXA-72 in Serbia; OXA-23 in Albania; and OXA-23, OXA-58, and OXA-72 CPs in Croatia, Serbia, and Bosnia and Herzegovina.

The molecular typing of *A. baumannii* isolates revealed the presence of four distinct clusters: the ST2 cluster was found in isolates from Bucharest, indicating a localized prevalence in this region, while the cluster containing the ST10 clone was primarily associated with isolates from Târgoviște, suggesting a regional specificity for this sequence type. *A. baumannii* ST10 has been found in clinical and community-acquired infections globally, including USA [54], Vietnam [55], Iran [56], Australia [57], Belgium [58], and Germany [59]. Moreover, pangenome analysis demonstrated that the genomes of *A. baumannii* are open, as indicated by Heaps’ law (γ = 0.26). An open genome indicates that the gene pool of an isolate has not reached an upper limit, thus allowing the acquisition of new genes through transposable elements [60]. This finding was corroborated by Gherghe-Barbu and collaborators, who used the same tool (e.g., Seth Commichaux’s Python script) to analyze the pangenome of *A. baumannii* isolated from WW and clinical samples in Târgoviște and Ramnicu Valcea, where the value was γ = 0.41 [10].

Further, our study aimed to quantify *Acinetobacter* species in water samples. Metagenomic reads corresponding to Moraxellaceae family members were found at less than 1% in UP (with no reads assigned to *Acinetobacter*), 2% in the DO of Târgoviște, and 6% in the DO of Glina. This indicates that wastewater contributes to the enrichment of surface waters with Moraxellaceae family bacteria. In DO samples, *Acinetobacter* constituted approximately half of the Moraxellaceae reads (43% in Glina and 52% in Târgoviște), suggesting a significant impact of urban wastewater on microbial communities. However, culture-dependent methods revealed the presence of *Acinetobacter* in UP samples, indicating that *Acinetobacter* exists in UP samples at very low abundances, below the detection threshold of 16S metagenomic sequencing.

Regarding the microbial composition of water samples, we observed that the low abundance of Alpha Proteobacteria and Actinobacteria in both Târgoviște and Glina, Bucharest, samples contrasts with the typical composition of aquatic microbiota [61]. This deviation could be indicative of specific environmental pressures or contamination events affecting these communities. Factors such as pollution, nutrient loads, or other anthropogenic activities could be influencing the microbial balance.

The dominance of certain bacterial orders in UP versus DO samples highlights the impact of local environmental conditions and potential sources of contamination. The presence of pathogenic bacteria like Campylobacteraceae in both locations underscores public health concerns, especially regarding the use of these water bodies for recreational or agricultural purposes. The elevated levels of *Enterobacteriaceae* and other human-associated bacteria in DO samples suggest fecal contamination, likely from sewage discharge or runoff from agricultural lands. The presence of pathogenic bacteria like Campylobacteraceae indicates a risk of waterborne diseases, necessitating stringent water quality monitoring and management strategies [62].

Lastly, our study aimed to determine the presence of *Acinetobacter* spp. in the microbiota of *Carassius auratus*, sampled in the DO regions of both WWTPs. *Carassius gibelio* is one of the most prevalent fish species in Romanian lakes and is inevitably exposed to various pollutants in the water. Being omnivorous, these fish feed on a diverse diet that includes plankton, invertebrates, plant material, and detritus. Studies show that Proteobacteria are typically one of the most dominant phyla in the gut microbiome of *Carassius gibelio* [63]. Bacteroidetes are also present, though often in lower abundance compared to Proteobacteria and Firmicutes, whereas Fusobacteria are particularly abundant in older fish. The main genera reporter in the *Carrasius* gut microbiota are *Aeromonas*, *Pseudomonas*, *Acinetobacter*, *Shewanella*, and *Serratia* [63]. Similar to the study by Li et al., we identified Proteobacteria as the dominant phyla and genera such as *Aeromonas*, *Shewanella*, and *Pseudomonas* as part of the *Carassius gibelio* microbiome.

The fish samples from Târgoviște and Glina, Bucharest, exhibited distinct microbial profiles, reflecting differences in environmental conditions and potential contamination sources at these sites. The differences in microbial diversity and composition may be attributed to varying levels of pollution and human impact. Glina’s lower microbial diversity and higher presence of Enterobacteriaceae suggest more significant environmental stress and contamination, as well as fecal pollution.

The combined analysis of 16S rRNA metagenomic data and chromogenic culture media findings underscores the significant presence of *Acinetobacter* in surface water samples from the DO region of Glina, Bucharest, and Târgoviște WWTPs. This high prevalence is associated with elevated microbial loads and significant resistance phenotypes (CARBA, ESBL, and colistin), especially in the receiving river from DO regions. These insights are important for developing strategies to monitor and mitigate the spread of ARB in the environment, ensuring public health safety and effective wastewater treatment practices.

Limitations of this study may arise from the fact that the samples were collected at a single point in time, which does not account for seasonal or temporal variations in microbial load and resistance patterns, which could affect the generalizability of the findings, as well as the fact that this study did not extensively analyze environmental factors such as water temperature, pH, or nutrient levels, which could influence the microbial communities and antibiotic resistance patterns.

## 5. Conclusions

Our study tracked the transmission of *A. baumannii* from hospital to aquatic ecosystems in southern Romania. We revealed that, although we encountered MDR isolates, some belonging to widely disseminated clones, the amount of *Acinetobacter* sp. in DO waters is very low and our data suggest that it is not acquired by the most abundant fish species (*C. auratus*) present in the respective waters.

On the other hand, the findings further underscore WWTPs as reservoirs for MDR *A. baumannii*, highlighting the potential for environmental dissemination and public health risks. Deviations in microbial community composition, both in surface waters and in *C. auratus* microbiota, suggest specific environmental pressures, necessitating stringent water quality monitoring and integrated surveillance strategies to mitigate public health risks associated with fecal contamination and pathogenic bacteria.

## Figures and Tables

**Table 1:** MIC values for colistin susceptibility in clinical and wastewater *A. baumannii* isolates from southern Romania.

Antibiotic/ Isolate	22012-CA5	22012-ENE6	22013-CA5	22013-ENE4	22014-CA2	22014-COLN5	22015-CA3	22015-CA4	22015-ENE6	22016-CA2	22016-CNE3	22016-CNE4	22017-CNE1	22018-CA5	22018-CA6	22019-CNE4	22019-CNE5	24-IHI	3-IHI	49-IHI
Colistin (µG/ML)	0.25	<0.25	<0.25	<0.25	<0.25	<0.25	<0.25	<0.25	<0.25	<0.25	<0.25	<0.25	<0.25	<0.25	<0.25	<0.25	<0.25	1	<0.25	<0.25

## Data Availability

*A. baumannii* isolates and data sets regarding the results are available from the authors.

## Supplementary Materials

The following supporting information can be downloaded at: https://www.mdpi.com/article/10.3390/microorganisms12081703/s1. Figure S1: Krona plots illustrating microbial community composition based on 16S rRNA sequencing of fish intestine samples from Glina, Bucharest, and Târgoviște. (A) Krona plot representing microbial taxa present in fish intestines from Glina, Bucharest. (B) Krona plot representing microbial taxa present in fish intestines from Târgoviște. Each segment in the plots represents a taxonomic group at different levels (phylum, class, order, etc.), with the size of the segments corresponding to the relative abundance of that taxon within the sample. Taxonomic labels are color-coded for clarity. Figure S2: Krona plots illustrating microbial community composition based on 16S rRNA sequencing of upstream and downstream water samples in Târgoviște (A) and Glina (B). Table S1: *A. baumannii* isolates from Bucharest wastewater, surface water, and intra-hospital infection samples. Table S2: *A. baumannii* isolates from Târgoviște wastewater and surface water samples. Table S3: log_10_ CFU/100 mL in surface water samples. Table S4: log_10_ CFU/100 mL in wastewater samples. Table S5: Antimicrobial resistance profiles of *A. baumannii* isolated from Bucharest WWTP and Fundeni Hospital in 2022. Table S6: Antimicrobial resistance profiles of *A. baumannii* isolated from Târgoviște WWTP in 2022. Table S7: Genetic support for AR of *A. baumannii* isolated from Bucharest WWTP and Fundeni Hospital in 2022. Table S8: Genetic support for the AR of *A. baumanii* isolated from Târgoviște WWTP in 2022. Table S9: ARGs profiles for *A. baumannii* isolated from WWTPs and Fundeni Hospital in Romania. Table S10: Virulence factors profiles for *A. baumannii* isolated from intra-hospital infections and WWTPs in Romania.

## References

[1] Drane K., Sheehan M., Whelan A., Ariel E., Kinobe R. (2024). The Role of Wastewater Treatment Plants in Dissemination of Antibiotic Resistance: Source, Measurement, Removal and Risk Assessment. Antibiotics.

[2] Calistri P., Iannetti S.L., Danzetta M., Narcisi V., Cito F., Di Sabatino D., Bruno R., Sauro F., Atzeni M., Carvelli A. (2013). The Components of “One World—One Health” Approach. Transbound. Emerg. Dis..

[3] Amos G.C.A., Ploumakis S., Zhang L., Hawkey P.M., Gaze W.H., Wellington E.M.H. (2018). The Widespread Dissemination of Integrons throughout Bacterial Communities in a Riverine System. ISME J..

[4] Pérez-Valera E., de Melo Rangel W., Elhottová D. (2022). Cattle Manure Application Triggers Short-Term Dominance of Acinetobacter in Soil Microbial Communities. Appl. Soil Ecol..

[5] Barbu I.C., Gheorghe-Barbu I., Grigore G.A., Vrancianu C.O., Chifiriuc M.C. (2023). Antimicrobial Resistance in Romania: Updates on Gram-Negative ESCAPE Pathogens in the Clinical, Veterinary, and Aquatic Sectors. Int. J. Mol. Sci..

[6] Rosenberg Goldstein R.E., Micallef S.A., Gibbs S.G., George A., Claye E., Sapkota A., Joseph S.W., Sapkota A.R. (2014). Detection of Vancomycin-Resistant Enterococci (VRE) at Four U.S. Wastewater Treatment Plants That Provide Effluent for Reuse. Sci. Total Environ..

[7] Poirel L., Naas T., Nordmann P. (2010). Diversity, Epidemiology, and Genetics of Class D β-Lactamases. Antimicrob. Agents Chemother..

[8] Blanco N., Harris A.D., Rock C., Johnson J.K., Pineles L., Bonomo R.A., Srinivasan A., Pettigrew M.M., Thom K.A. (2018). Risk Factors and Outcomes Associated with Multidrug-Resistant *Acinetobacter Baumannii* upon Intensive Care Unit Admission. Antimicrob. Agents Chemother..

[9] https://atlas.ecdc.europa.eu/public/index.aspx.

[10] Gheorghe-Barbu I., Surleac M., Barbu I.C., Paraschiv S., Bănică L.M., Rotaru L.I., Vrâncianu C.O., Niță Lazăr M., Oțelea D., Chifiriuc M.C. (2024). Decoding the Resistome, Virulome and Mobilome of Clinical versus Aquatics *Acinetobacter baumannii* in Southern Romania. Heliyon.

[11] Gheorghe-Barbu I., Corbu V.M., Vrancianu C.O., Marinas I.C., Popa M., Dumbravă A.S., Niță-Lazăr M., Pecete I., Muntean A.A., Popa M.I. (2023). Phenotypic and Genotypic Characterization of Recently Isolated Multidrug-Resistant *Acinetobacter baumannii* Clinical and Aquatic Strains and Demonstration of Silver Nanoparticle Potency. Microorganisms.

[12] Gheorghe I., Cristea V.C., Marutescu L., Popa M., Murariu C., Trusca B.S., Borcan E., Ghita C., Lazar V., Chifiriuc M.C. (2019). Resistance and Virulence Features in Carbapenem-Resistant *Acinetobacter baumannii* Community Acquired and Nosocomial Isolates in Romania. Rev. Chim..

[13] Vrâncianu C.O., Gheorghe-Barbu I., Czobor Barbu I., Mãruþescu L., Popa M., Niþã-Lazãr M., Muntean A.-A., Dragomirescu C., Sãndulescu O., Talapan D. (2023). Antibiotic resistantance profiles in *Acinetobacter baumannii* strains isolates from wastewater in sourthen Romania. Rom. Arch. Microbiol. Immunol..

[14] Ma C., McClean S. (2021). Mapping Global Prevalence of *Acinetobacter baumannii* and Recent Vaccine Development to Tackle It. Vaccines.

[15] Dekić S., Klobučar G., Ivanković T., Zanella D., Vucić M., Bourdineaud J.-P., Hrenović J. (2018). Emerging Human Pathogen *Acinetobacter baumannii* in the Natural Aquatic Environment: A Public Health Risk?. Int. J. Environ. Health Res..

[16] https://www.aqualia.com/en/web/aqualia-global/-/aqualia-and-fccco-complete-the-expansion-of-glina-wwtp-bucharest-romania-.

[17] https://www.hillintl.com/project/bucharest-glina-wastewater-treatment-plant-phase-ii/.

[18] https://www.aquastrategy.com/article/romanian-wastewater-treatment-contract-award.

[19] https://www.erbasu.ro/en/proiect/wastewater-treatment-plants-targoviste/.

[20] https://www.parcis.eu/portfolio/targoviste-wwtp-dambovita-county-romania/.

[21] CLSI (2022). Performance Standards for Antimicrobial Susceptibility Testing.

[22] https://github.com/tseemann/shovill.

[23] Seemann T. (2014). Prokka: Rapid Prokaryotic Genome Annotation. Bioinformatics.

[24] https://github.com/tseemann/abricate.

[25] Chen L., Zheng D., Liu B., Yang J., Jin Q. (2016). VFDB 2016: Hierarchical and Refined Dataset for Big Data Analysis—10 Years On. Nucleic Acids Res..

[26] Seemann T. (2024). Mlst.

[27] Page A.J., Cummins C.A., Hunt M., Wong V.K., Reuter S., Holden M.T.G., Fookes M., Falush D., Keane J.A., Parkhill J. (2015). Roary: Rapid Large-Scale Prokaryote Pan Genome Analysis. Bioinformatics.

[28] Hadfield J., Croucher N.J., Goater R.J., Abudahab K., Aanensen D.M., Harris S.R. (2018). Phandango: An Interactive Viewer for Bacterial Population Genomics. Bioinformatics.

[29] https://github.com/kwongj/roary2fripan.

[30] https://drpowell.github.io/FriPan/.

[31] https://github.com/SethCommichaux/Heap_Law_for_Roary.

[32] Jovcic B., Novovic K., Dekic S., Hrenovic J. (2021). Colistin Resistance in Environmental Isolates of *Acinetobacter baumannii*. Microb. Drug Resist..

[33] Shelenkov A., Akimkin V., Mikhaylova Y. (2023). International Clones of High Risk of *Acinetobacter baumannii*—Definitions, History, Properties and Perspectives. Microorganisms.

[34] http://www.mgc.ac.cn/cgi-bin/VFs/genus.cgi?Genus=Acinetobacter.

[35] Zaharia L., Ioana-Toroimac G., Cocoş O., Ghiţă F.A., Mailat E. (2016). Urbanization Effects on the River Systems in the Bucharest City Region (Romania). Ecosyst. Health Sustain..

[36] Manoiu V.-M. (2017). Water Quality Changes in Ialomiţa River under the Influence of Human Settlements Activities. Aerul Apa Compon. Mediu..

[37] Novović K., Jovčić B. (2023). Colistin Resistance in *Acinetobacter baumannii*: Molecular Mechanisms and Epidemiology. Antibiotics.

[38] Ušjak D., Novović K., Filipić B., Kojić M., Filipović N., Stevanović M.M., Milenković M.T. (2022). In Vitro Colistin Susceptibility of Pandrug-resistant *Acinetobacter baumannii* Is Restored in the Presence of Selenium Nanoparticles. J. Appl. Microbiol..

[39] Cafiso V., Stracquadanio S., Lo Verde F., Gabriele G., Mezzatesta M.L., Caio C., Pigola G., Ferro A., Stefani S. (2019). Colistin Resistant *Acinetobacter baumannii*: Genomic and Transcriptomic Traits Acquired Under Colistin Therapy. Front. Microbiol..

[40] Kabic J., Novovic K., Kekic D., Trudic A., Opavski N., Dimkic I., Jovcic B., Gajic I. (2023). Comparative Genomics and Molecular Epidemiology of Colistin-Resistant *Acinetobacter baumannii*. Comput. Struct. Biotechnol. J..

[41] Mustapha M.M., Li B., Pacey M.P., Mettus R.T., McElheny C.L., Marshall C.W., Ernst R.K., Cooper V.S., Doi Y. (2018). Phylogenomics of Colistin-Susceptible and Resistant XDR *Acinetobacter baumannii*. J. Antimicrob. Chemother..

[42] Trebosc V., Gartenmann S., Tötzl M., Lucchini V., Schellhorn B., Pieren M., Lociuro S., Gitzinger M., Tigges M., Bumann D. (2019). Dissecting Colistin Resistance Mechanisms in Extensively Drug-Resistant *Acinetobacter baumannii* Clinical Isolates. mBio.

[43] Fam N.S., Gamal D., Mohamed S.H., Wasfy R.M., Soliman M.S., El-Kholy A.A., Higgins P.G. (2020). Molecular Characterization of Carbapenem/Colistin-Resistant *Acinetobacter baumannii* Clinical Isolates from Egypt by Whole-Genome Sequencing. Infect. Drug Resist..

[44] Palmieri M., D’Andrea M.M., Pelegrin A.C., Perrot N., Mirande C., Blanc B., Legakis N., Goossens H., Rossolini G.M., van Belkum A. (2020). Abundance of Colistin-Resistant, OXA-23- and ArmA-Producing *Acinetobacter baumannii* Belonging to International Clone 2 in Greece. Front. Microbiol..

[45] Thadtapong N., Chaturongakul S., Soodvilai S., Dubbs P. (2021). Colistin and Carbapenem-Resistant *Acinetobacter baumannii* Aci46 in Thailand: Genome Analysis and Antibiotic Resistance Profiling. Antibiotics.

[46] Pournaras S., Poulou A., Dafopoulou K., Chabane Y.N., Kristo I., Makris D., Hardouin J., Cosette P., Tsakris A., Dé E. (2014). Growth Retardation, Reduced Invasiveness, and Impaired Colistin-Mediated Cell Death Associated with Colistin Resistance Development in *Acinetobacter baumannii*. Antimicrob. Agents Chemother..

[47] Agodi A., Voulgari E., Barchitta M., Quattrocchi A., Bellocchi P., Poulou A., Santangelo C., Castiglione G., Giaquinta L., Romeo M.A. (2014). Spread of a Carbapenem- and Colistin-Resistant *Acinetobacter baumannii* ST2 Clonal Strain Causing Outbreaks in Two Sicilian Hospitals. J. Hosp. Infect..

[48] Lowe M., Singh-Moodley A., Ismail H., Thomas T., Chibabhai V., Nana T., Lowman W., Ismail A., Chan W.Y., Perovic O. (2022). Molecular Characterisation of *Acinetobacter baumannii* Isolates from Bloodstream Infections in a Tertiary-Level Hospital in South Africa. Front. Microbiol..

[49] Nogbou N.-D., Ramashia M., Nkawane G.M., Allam M., Obi C.L., Musyoki A.M. (2022). Whole-Genome Sequencing of a Colistin-Resistant *Acinetobacter baumannii* Strain Isolated at a Tertiary Health Facility in Pretoria, South Africa. Antibiotics.

[50] Blejan I.E., Diaconu C.E., Arsene A.L., Udeanu D.I., Ghica M., Drăgănescu D., Dragomiroiu G.T.A.B., Rădulescu M., Maltezou H.C., Tsatsakis A.M. (2020). Antibiotic Resistance in Community-Acquired Pneumonia. A Romanian Perspective. Farmacia.

[51] Goic-Barisic I., Hrenovic J., Kovacic A., Musić M.Š. (2016). Emergence of Oxacillinases in Environmental Carbapenem-Resistant *Acinetobacter baumannii* Associated with Clinical Isolates. Microb. Drug Resist..

[52] Pulami D., Kämpfer P., Glaeser S.P. (2023). High Diversity of the Emerging Pathogen *Acinetobacter baumannii* and Other *Acinetobacter* spp. in Raw Manure, Biogas Plants Digestates, and Rural and Urban Wastewater Treatment Plants with System Specific Antimicrobial Resistance Profiles. Sci. Total Environ..

[53] Higgins P.G., Hrenovic J., Seifert H., Dekic S. (2018). Characterization of *Acinetobacter baumannii* from Water and Sludge Line of Secondary Wastewater Treatment Plant. Water Res..

[54] Jones C.L., Clancy M., Honnold C., Singh S., Snesrud E., Onmus-Leone F., McGann P., Ong A.C., Kwak Y., Waterman P. (2015). Fatal Outbreak of an Emerging Clone of Extensively Drug-Resistant *Acinetobacter baumannii* with Enhanced Virulence. Clin. Infect. Dis..

[55] Schultz M.B., Pham Thanh D., Tran Do Hoan N., Wick R.R., Ingle D.J., Hawkey J., Edwards D.J., Kenyon J.J., Phu Huong Lan N., Campbell J.I. (2016). Repeated Local Emergence of Carbapenem-Resistant *Acinetobacter baumannii* in a Single Hospital Ward. Microb. Genom..

[56] Abhari S.S., Badmasti F., Modiri L., Aslani M.M., Asmar M. (2019). Circulation of Imipenem-Resistant *Acinetobacter baumannii* ST10, ST2 and ST3 in a University Teaching Hospital from Tehran, Iran. J. Med. Microbiol..

[57] Meumann E.M., Anstey N.M., Currie B.J., Piera K.A., Kenyon J.J., Hall R.M., Davis J.S., Sarovich D.S. (2019). Genomic Epidemiology of Severe Community-Onset *Acinetobacter baumannii* Infection. Microb. Genom..

[58] Valcek A., Nesporova K., Whiteway C., De Pooter T., De Coster W., Strazisar M., Van der Henst C. (2022). Genomic Analysis of a Strain Collection Containing Multidrug, Extensively Drug, Pandrug, and Carbapenem-Resistant Modern Clinical Isolates of *Acinetobacter baumannii*. Antimicrob. Agents Chemother..

[59] Savin M., Sib E., Heinemann C., Eichel V.M., Nurjadi D., Klose M., Andre Hammerl J., Binsker U., Mutters N.T. (2024). Tracing Clinically-Relevant Antimicrobial Resistances in *Acinetobacter baumannii*-Calcoaceticus Complex across Diverse Environments: A Study Spanning Clinical, Livestock, and Wastewater Treatment Settings. Environ. Int..

[60] Park S.C., Lee K., Kim Y.O., Won S., Chun J. (2019). Large-Scale Genomics Reveals the Genetic Characteristics of Seven Species and Importance of Phylogenetic Distance for Estimating Pan-Genome Size. Front. Microbiol..

[61] Sehnal L., Brammer-Robbins E., Wormington A.M., Blaha L., Bisesi J., Larkin I., Martyniuk C.J., Simonin M., Adamovsky O. (2021). Microbiome Composition and Function in Aquatic Vertebrates: Small Organisms Making Big Impacts on Aquatic Animal Health. Front. Microbiol..

[62] Shayo G.M., Elimbinzi E., Shao G.N., Fabian C. (2023). Severity of Waterborne Diseases in Developing Countries and the Effectiveness of Ceramic Filters for Improving Water Quality. Bull. Natl. Res. Cent..

[63] Li X., Zhou L., Yu Y., Li Y., Zhao T., Feng W., Luo K., Yu Y. (2017). Composition of Gut Microbiota in the Gibel Carp (*Carassius auratus gibelio*) Varies with Host Development. Microb. Ecol..

