# Supplementary material for: Tracing Acinetobacter baumannii’s Journey from Hospitals to Aquatic Ecosystems
Source: Microorganisms. 2024 Aug 18;12(8):1703. doi: 10.3390/microorganisms12081703 (PMC11356923; doi:10.3390/microorganisms12081703)
Supplement: Supplementary file 1 [file microorganisms-12-01703-s001.zip › microorganisms-3148404-supplementary.pdf]

microorganisms

MDPI

Supplementary Material

Tracing *Acinetobacter baumannii*'s Journey from Hospitals to Aquatic Ecosystems

Irina Gheorghe-Barbu [1,2,†], Rares-Ionut Dragomir [1,2,†], Gratiela Gradisteanu Pircalabioru [1,2,*], Marius Surleac [2,3], Iulia Adelina Dinu [1], Madalina Diana Gaboreanu [1,2] and Ilda Czobor Barbu [1,2]

[1] Faculty of Biology, University of Bucharest, Intr. Portocalelor No. 1–3, 060101 Bucharest, Romania; irina.gheorghe@bio.unibuc.ro (I.G.-B.); r.dragomir20@s.bio.unibuc.ro (R.-I.D.); dinu.iulia-adelina@s.bio.unibuc.ro (I.A.D.); gaboreanu.diana-madalina@s.bio.unibuc.ro (M.D.G.); ilda.barbu@bio.unibuc.ro (I.C.B.)

[2] The Research Institute of the University of Bucharest (ICUB), B.P Hasdeu No. 7, 050095 Bucharest, Romania; marius.surleac@gmail.com

[3] National Institute for Infectious Diseases, "Matei Balș", Dr. Calistrat Grozovici No. 1, 021105 Bucharest, Romania

* Correspondence: gratiela.gradisteanu@icub.unibuc.ro

† These authors contributed equally to this work

Supplementary Figures

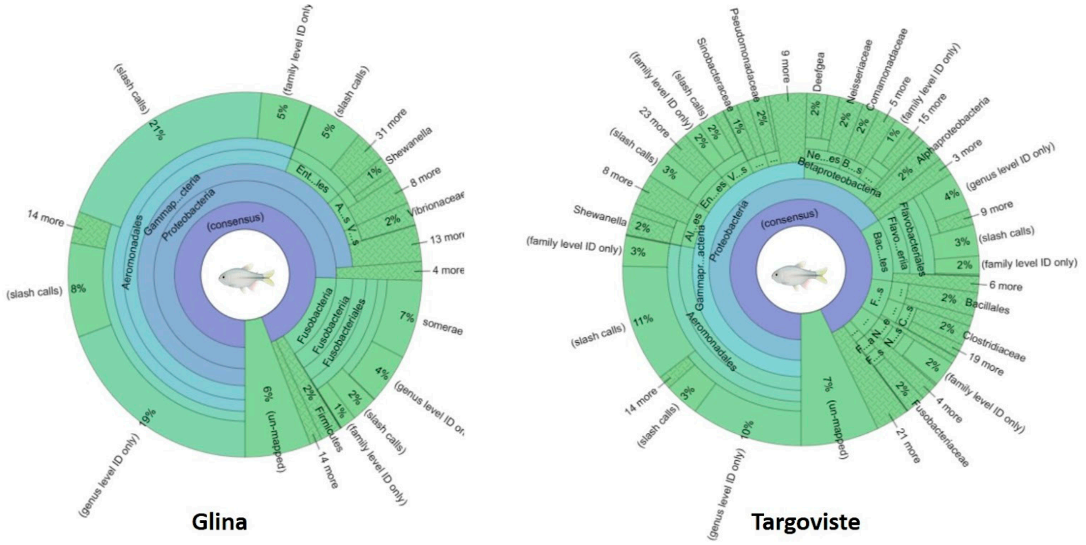

Figure S1. Krona plots illustrating microbial community composition based on 16S rRNA sequencing of fish intestine samples from Glina, Bucharest and Targoviste. (A) Krona plot representing microbial taxa present in fish intestines from Glina, Bucharest. (B) Krona plot representing microbial taxa present in fish intestines from Targoviste. Each segment in the plots represents a taxonomic group at different levels (phylum, class, order, etc.), with the size of the segments corresponding to the relative abundance of that taxon within the sample. Taxonomic labels are color-coded for clarity.

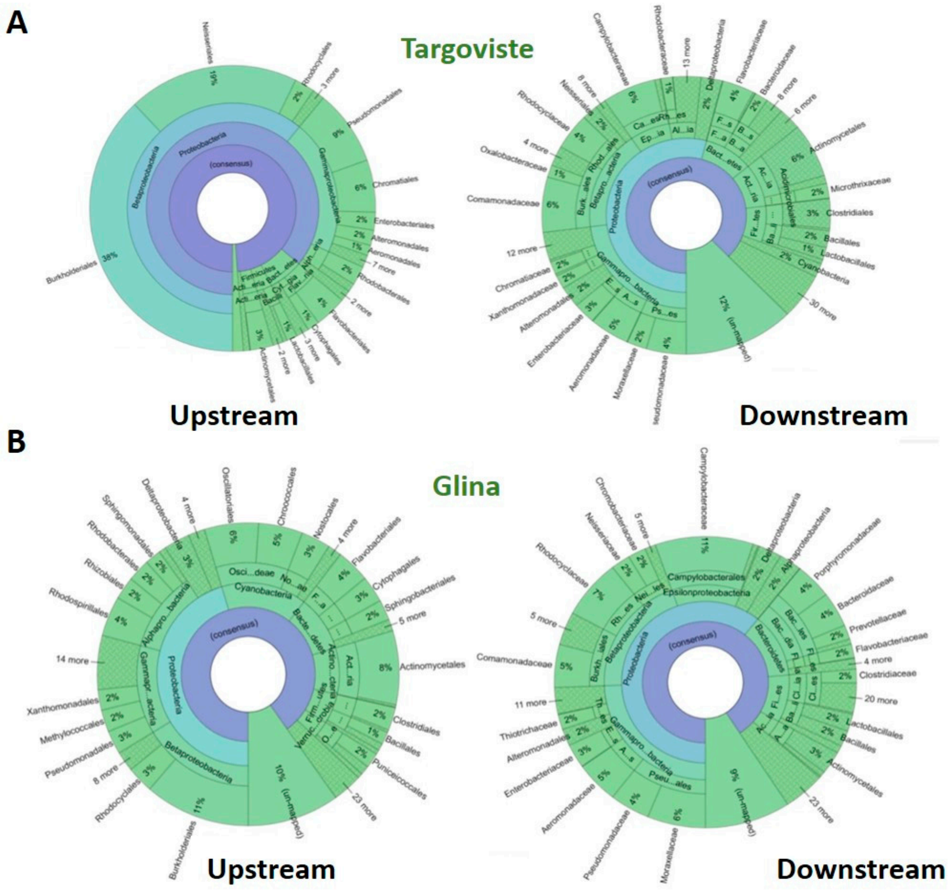

Figure S2. Krona plots illustrating microbial community composition based on 16S rRNA sequencing of upstream and downstream water samples in Targoviste (A) and Glina (B).

Supplementary Tables

Supplementary Table S1. *A. baumannii* isolates from Bucharest wastewater, surface water and intra-hospital infection samples.

Strain code	*Isolation source*	*Culture media /Isolation source*
22012 ENE 6	surface water – downstream river	CHROMagar ESBL
22012 ENE 3	surface water – downstream river	CHROMagar ESBL
22012 ENE 5	surface water – downstream river	CHROMagar ESBL
22012 CA 6	surface water – downstream river	CHROMagar Acinetobacter
22012 CA 1	surface water – downstream river	CHROMagar Acinetobacter
22012 CA 4	surface water – downstream river	CHROMagar Acinetobacter
22012 CA 5	surface water – downstream river	CHROMagar Acinetobacter
22013 ENE 4	WWTP - influent	CHROMagar ESBL
22013 ENE 1	WWTP - influent	CHROMagar ESBL
22013 CA4	WWTP - influent	CHROMagar Acinetobacter
22013 CA5	WWTP - influent	CHROMagar Acinetobacter
22013 CA1	WWTP - influent	CHROMagar Acinetobacter

22013 CA2	WWTP - influent	CHROMagar Acinetobacter
22013 CA3	WWTP - influent	CHROMagar Acinetobacter
22014 ENE 4	WWTP - effluent	CHROMagar ESBL
22014 ENE 5	WWTP - effluent	CHROMagar ESBL
22014 ENE 2	WWTP - effluent	CHROMagar ESBL
22014 ENE 3	WWTP - effluent	CHROMagar ESBL
22014 CA 4	WWTP - effluent	CHROMagar Acinetobacter
22014 CA 5	WWTP - effluent	CHROMagar Acinetobacter
22014 CA 6	WWTP - effluent	CHROMagar Acinetobacter
22014 CA 1	WWTP - effluent	CHROMagar Acinetobacter
22014 CA 2	WWTP - effluent	CHROMagar Acinetobacter
22014 CA 3	WWTP - effluent	CHROMagar Acinetobacter
22014 COL N 5	WWTP - effluent	CHROMagar Colistin
22015 COL N 2	WWTP – active sludge	CHROMagar Colistin
22015 ENE 6	WWTP – active sludge	CHROMagar ESBL
22015 ENE 1	WWTP – active sludge	CHROMagar ESBL
22015 ENE 4	WWTP – active sludge	CHROMagar ESBL
22015 CA 4	WWTP – active sludge	CHROMagar Acinetobacter
22015 CA 1	WWTP – active sludge	CHROMagar Acinetobacter
22015 CA 3	WWTP – active sludge	CHROMagar Acinetobacter
22015 CA 2	WWTP – active sludge	CHROMagar Acinetobacter
Intra-hospital infections Fundeni Hospital	1 Abc	rectal swab
	2 Abc	rectal swab
	3 Abc	bronchial secretion
	4 Abc	bronchial secretion
	5 Abc	plague secretion
	6 Abc	bronchial secretion
	7 Abc	bronchial secretion
	8 Abc	plague secretion
	9 Abc	plague secretion
	10 Abc	bronchial secretion
	11 Abc	bronchial secretion
	12 Abc	Rectal swab
	13 Abc	bronchial secretion
	14 Abc	peritoneal fluid
	24 Fundeni	rectal swab
	3 Fundeni	rectal swab
	49 Fundeni	rectal swab

Supplementary Table S2. *A. baumannii* isolates from Targoviste wastewater and surface water samples.

STRAIN CODE	ISOLATION SOURCE	CULTURE MEDIA
22016 COL N6	surface water-upstream	CHROMagar Colistin
22016 CNE 1	surface water-upstream	CHROMagar CARBA

22016 CNE 2	surface water-upstream	CHROMagar CARBA
22016 CNE 3	surface water-upstream	CHROMagar CARBA
22016 CNE 4	surface water-upstream	CHROMagar CARBA
22016 ENE 4	surface water-upstream	CHROMagar ESBL
22016 CA 1	surface water-upstream	CHROMagar Acinetobacter
22016 CA 2	surface water-upstream	CHROMagar Acinetobacter
22016 CA 3	surface water-upstream	CHROMagar Acinetobacter
22016 CA 4	surface water-upstream	CHROMagar Acinetobacter
22016 CA 5	surface water-upstream	CHROMagar Acinetobacter
22016 CA 6	surface water-upstream	CHROMagar Acinetobacter
22017 CNE 1	surface water-downstream	CHROMagar CARBA
22017 CNE 2	surface water-downstream	CHROMagar CARBA
22017 CNE 3	surface water-downstream	CHROMagar CARBA
22017 CA 1	surface water-downstream	CHROMagar Acinetobacter
22017 CA 2	surface water-downstream	CHROMagar Acinetobacter
22017 CA 3	surface water-downstream	CHROMagar Acinetobacter
22017 CA 4	surface water-downstream	CHROMagar Acinetobacter
22017 CA 5	surface water-downstream	CHROMagar Acinetobacter
22018 CA 1	WWTP-influent	CHROMagar Acinetobacter
22018 CA 2	WWTP-influent	CHROMagar Acinetobacter
22018 CA 3	WWTP-influent	CHROMagar Acinetobacter
22018 CA 4	WWTP-influent	CHROMagar Acinetobacter
22018 CA 5	WWTP-influent	CHROMagar Acinetobacter
22018 CA 6	WWTP-influent	CHROMagar Acinetobacter
22019 CA 4 TARGOV	WWTP-effluent	CHROMagar Acinetobacter
22019 CNE 4	WWTP-effluent	CHROMagar CARBA
22019 CNE 5	WWTP-effluent	CHROMagar CARBA
22019 CNE 6	WWTP-effluent	CHROMagar CARBA
22019 CNE 1	WWTP-effluent	CHROMagar CARBA
22019 CNE 2	WWTP-effluent	CHROMagar CARBA
22019 CNE 3	WWTP-effluent	CHROMagar CARBA

Supplementary Table S3. log_{10} CFU/100 mL in surface water samples.

Isolation source/ Phenotype	Acinetobacter phenotype	ESBL phenotype	CARBA phenotype	Colistin phenotype
**Upstream WWTP Bucharest – Dâmbovița River**	2.104	2.597	2.480	2.602
**Upstream WWTP Târgoviște – Ialomița River**	2.524	2.602	2.602	1.477
**Downstream WWTP Bucharest – Dâmbovița River**	3.199	4.363	3.268	4.091

**Downstream WWTP Târgoviște – Ialomița River**	2.671	2.754	2.241	4.823

Supplementary Table S4. log_{10} CFU/100 mL in wastewater samples.

Isolation source/ Phenotype	Acinetobacter phenotype	ESBL phenotype	CARBA phenotype
**Bucharest WWTP Influent**	4.740	4.852	3.910
**Târgoviște WWTP Influent**	4.112	3.686	3.065
**Bucharest WWTP Active sludge**	3.699	3.446	2.826
**Bucharest WWTP Effluent**	3.270	5.074	4.141
**Târgoviște WWTP Effluent**	2.037	2.831	2.371

Supplementary Table S5. Antimicrobial resistance profiles of *A. baumannii* isolated from Bucharest WWTP and Fundeni Hospital in 2022.

Isolation source/antibiotics	β-lactams (%)	Fluoroquinolones (%)	Aminoglycosides (%)	Tetracyclines (%)
**Intra-hospital Infections**	98	100	94	94
**WWTP Influent**	25	14	43	0
**WWTP Active sludge**	31	29	57	14
**WWTP Effluent**	21	9	36	18
**WWTP Downstream**	31	14	43	0

Supplementary Table S6. Antimicrobial resistance profiles of *A. baumannii* isolated from Targoviște WWTP in 2022.

Isolation source/antibiotics	β-lactams (%)	Fluoroquinolones (%)	Aminoglycosides (%)	Tetracyclines (%)
**WWTP Upstream**	42	17	54	0
**WWTP Influent**	33	33	50	13
**WWTP Effluent**	3	0	36	14
**WWTP Downstream**	29	0	50	0

Supplementary Table S7. Genetic support for AR of *A. baumannii* isolated from Bucharest WWTP and Fundeni Hospital in 2022.

Isolation source/gene	*bla*OXA-23 (%)	*bla*OXA-24 (%)	*bla*OXA-51 (%)	*bla*TEM (%)
Intra-hospital Infections	62	46	62	15
WWTP Influent	14	0	100	14
WWTP Active sludge	28	0	100	0
WWTP Effluent	19	9	100	0
WWTP Downstream	14	14	100	0

Supplementary Table S8. Genetic support for the AR of *A. baumanii* isolated from Targoviște WWTP in 2022.

Isolation source/gene	*bla*OXA-51 (%)	*bla*CTX-M (%)
WWTP Upstream	100	0
WWTP Influent	100	0
WWTP Effluent	100	0
WWTP Downstream	100	13

Supplementary Table S9. ARGs profiles for *A. baumannii* isolated from WWTPs and Fundeni Hospital in Romania

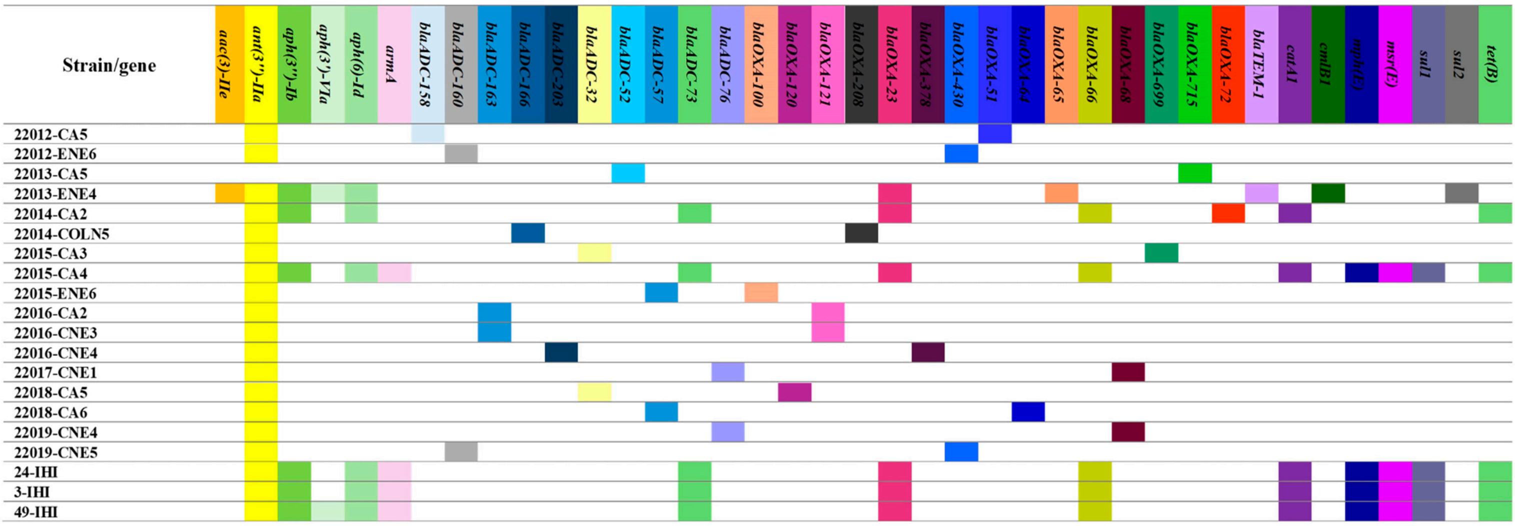

Supplementary Table S10. Virulence factors profiles for *A. baumannii* isolated intra-hospital infections and WWTPs in Romania.

Strain/gene	*abaI*	*abaR*	*ACICU_RS00400*	*ACICU_RS00405*	*ACICU_RS00455*	*ACICU_RS00470*	*ACICU_RS00475*	*ACICU_RS00485*	*ACICU_RS04565*	*ACICU_RS04570*	*ACICU_RS04575*	*ACICU_RS04580*	*ACICU_RS04585*	*ACICU_RS04590*	*ACICU_RS04595*	*ACICU_RS04605*	*ACICU_RS04610*	*algW*	*ata*	*bap*	*bauA*	*clpV/tssH*	*cpaA*	*cps4I*	*csuA*	*csuA/B*	*csuB*	*csuC*	*csuD*	*csuE*	*galE*	*galU*	*hcp/tssD*	*hemO*	*pseB*	*pseC*	*pseF*	*pseG*	*pseH*	*pseI*	*tagX*	*tse2*	*tse3*	*tssA*	*tssB*	*tssC*	*tssE*	*tssF*	*tssG*	*tssK*	*tssL*	*tssM*	*tufA*	*tviB*	*wbpD*
**22012-CA5**																																																							
**22012-ENE6**																																																							
**22013-CA5**																																																							
**22013-ENE4**																																																							
**22014-CA2**																																																							
**22014-COLN5**																																																							
**22015-CA3**																																																							
**22015-CA4**																																																							
**22015-ENE6**																																																							
**22016-CA2**																																																							
**22016-CNE3**																																																							
**22016-CNE4**																																																							
**22017-CNE1**																																																							
**22018-CA5**																																																							
**22018-CA6**																																																							
**22019-CNE4**																																																							
**22019-CNE5**																																																							
**24-IHI**																																																							
**3-IHI**																																																							
**49-IHI**																																																							
